# Development of ESTs from chickpea roots and their use in diversity analysis of the *Cicer *genus

**DOI:** 10.1186/1471-2229-5-16

**Published:** 2005-08-17

**Authors:** Hutokshi K Buhariwalla, Jayashree B, K Eshwar, Jonathan H Crouch

**Affiliations:** 1 c/o JH. Crouch, Centro International de Mejoramiento de Maiz Y trigo (CIMMYT), Apdo. postal 6-641, 06600 Mexico, D.F., Mexico; 2 Centro International de Mejoramiento de Maiz Y trigo (CIMMYT), Apdo. postal 6-641, 06600 Mexico, D.F., Mexico; 3 International Crop Research Institute for the Semi-Arid Tropics (ICRISAT), Patancheru, Andhra Pradesh 502 324, India

## Abstract

**Background:**

Chickpea is a major crop in many drier regions of the world where it is an important protein-rich food and an increasingly valuable traded commodity. The wild annual *Cicer *species are known to possess unique sources of resistance to pests and diseases, and tolerance to environmental stresses. However, there has been limited utilization of these wild species by chickpea breeding programs due to interspecific crossing barriers and deleterious linkage drag. Molecular genetic diversity analysis may help predict which accessions are most likely to produce fertile progeny when crossed with chickpea cultivars. While, trait-markers may provide an effective tool for breaking linkage drag. Although SSR markers are the assay of choice for marker-assisted selection of specific traits in conventional breeding populations, they may not provide reliable estimates of interspecific diversity, and may lose selective power in backcross programs based on interspecific introgressions. Thus, we have pursued the development of gene-based markers to resolve these problems and to provide candidate gene markers for QTL mapping of important agronomic traits.

**Results:**

An EST library was constructed after subtractive suppressive hybridization (SSH) of root tissue from two very closely related chickpea genotypes (*Cicer arietinum*). A total of 106 EST-based markers were designed from 477 sequences with functional annotations and these were tested on *C. arietinum*. Forty-four EST markers were polymorphic when screened across nine *Cicer *species (including the cultigen). Parsimony and PCoA analysis of the resultant EST-marker dataset indicated that most accessions cluster in accordance with the previously defined classification of primary (*C. arietinum*, *C. echinospermum *and *C. reticulatum*), secondary (*C. pinnatifidum*, *C. bijugum *and *C. judaicum*), and tertiary (*C. yamashitae*, *C. chrossanicum *and *C. cuneatum*) gene-pools. A large proportion of EST alleles (45%) were only present in one or two of the accessions tested whilst the others were represented in up to twelve of the accessions tested.

**Conclusion:**

Gene-based markers have proven to be effective tools for diversity analysis in *Cicer *and EST diversity analysis may be useful in identifying promising candidates for interspecific hybridization programs. The EST markers generated in this study have detected high levels of polymorphism amongst both common and rare alleles. This suggests that they would be useful for allele-mining of germplasm collections for identification of candidate accessions in the search for new sources of resistance to pests / diseases, and tolerance to abiotic stresses.

## Background

Chickpea (*Cicer arietium *L.) is one of the world's more important but less studied leguminous food crop with over 10 M ha grown across the Americas, the Mediterranean basin, East Africa, the Middle East, Asia and Australia [1]. As a grain legume it plays a significant role in the nutrition of the rural and urban poor in the developing world, as it provides a protein-rich supplement to cereal-based diets particularly of vegetarians and subsistence farmers who cannot afford meat. Despite its economic importance, chickpea productivity has been low because of yield losses to foliar and soil-borne fungal diseases (ascochyta blight, fusarium wilt and botrytis grey mould), insect pests (helicoverpapod borer) and abiotic stresses such as drought, cold and salinity. Sources of resistance and tolerance to these constraints exist in the wild *Cicer *germplasm yet remain largely unused by conventional breeding programs [2-5]. Three annual *Cicer *gene-pools have been defined based on the available hybridization reports, biochemical and molecular diversity analysis [6]: species within the primary gene-pool (*C. arietinum*, *C. reticulatum *and *C. echinospermum*) can be readily crossed usually generating fully fertile progeny; while species within the secondary gene-pool (*C. bijugum*, *C. pinnatifidum *and *C. judaicum*) can be successfully crossed with the cultigen *C. arietinum*, providing hybrid embryos are rescued. However, the progeny of crosses between primary and second gene-pools are frequently sterile; finally, species within the tertiary gene-pool (*C. cuneatum*, *C. chorassanicum*, *C. yamashitae *and others) have not yet been successfully crossed with the cultigen *Cicer arietinum*.

Cultivated chickpea has a relatively low level of diversity, due to a series of bottlenecks caused by restricted distribution of the wild progenitors and the founder effect associated with domestication [7]. In addition, chickpea breeding programs have limited themselves to a small number of cultivated genotypes having sources of biotic stress resistance and abiotic stress tolerance with little or no use of wild species [5]. This has resulted in limited durability of resistances to many of the major pests and diseases, and limited progress in abiotic stress tolerance breeding. Wild germplasm accessions held at the International Centre for Agricultural Research in Dry Areas (ICARDA) in Syria, the International Institute for the Semi-Arid Tropics (ICRISAT) in India and other genebanks in USA, Europe and Australia contain valuable sources of novel genetic variation for improvement of these traits [6]. The wild *Cicer *species from the secondary gene-pool (particularly *C. bijugum*, *C. pinnatifidum *and *C. judaicum*) are known to possess multiple sources of pest and disease resistance and tolerance to abiotic stresses including drought and cold [7,8].

Where species barriers can be overcome, molecular markers will facilitate rapid and efficient transfer of economically important traits into cultivated breeding pools. This will have the added advantage of broadening the narrow genetic base of this crop and thereby reducing its vulnerability to evolving pest and disease pressures, while simultaneously providing stable increases in yield far beyond the current 0.6% annual improvement [1].

Most types of molecular markers have been tested in chickpea including isozymes [9-12], restriction fragment length polymorphism (RFLP) [13,14] random amplified polymorphic DNA markers (RAPDs) [15,16] amplified fragment length polymorphisms (AFLPs) [17], sequence characterized amplified regions (SCARs) [18], inter-simple sequence repeat (ISSRs) [15], simple sequence repeat (STMS or SSR) [19-22], resistance gene analogs (RGAs) [23] DNA amplification fingerprinting (DAF) [24], and expressed sequence tags (ESTs) [25]. However, there is a low level of polymorphism detected in cultivated chickpea using isozyme/allozyme markers [9,10] and RFLP analysis [13,14]. In contrast, SSR markers have been shown to be highly polymorphic within the cultigen *C. arietinum *[26] and have been routinely utilized for creating genetic linkage maps [27-29]. These SSR markers have also been used as reference points for integration of the different chickpea linkage groups derived from inter- and intra- specific crosses [28,29]. To date there are over 500 SSR markers developed from chickpea genomic libraries [19,20,22]. In general, 30–50% of the chickpea SSR markers are polymorphic in any given breeding or intra-specific mapping population (HK Buhariwalla, unpublished data).

SSR markers remain the marker of choice for marker assisted selection in many breeding programs. However, SSR motifs may evolve too rapidly to be valuable as the sole assay for interspecific diversity analysis. In addition, markers shown to have tight genetic linkage to target genes in interspecific mapping populations may lose their selective power when used in backcross programs based on interspecific derivatives. Thus, there is a need for the development and utilization of gene-based markers that better serve molecular breeding applications and diversity analysis of germplasm.

In this context, the most important criteria for new markers are high reproducibility, detection of co-dominance polymorphism and suitability for rapid large-scale low cost screening. EST-based markers fulfill these criteria and since they are associated with the coding regions of the genome they also enhance molecular germplasm evaluation by capturing variation across transcribed regions and in genes of known function. This potentially resolves the problem of limited genomic coverage suffered by traditional single gene phylogenetic studies [30,31]. Targeted EST development has already begun in chickpea, focusing on ABA-related mechanisms of water-deficit tolerance in epicotyl tissue [32].

Our objective in this study was to increase the public EST resource for chickpea with a particular focus on root tissue, as constitutive drought tolerance mechanisms (such as root system development) are a common target for the crop physiology community [33] and are increasingly identified by genomics studies as important components of stress tolerance mechanisms [32,34]. Thus, an EST library was constructed after SSH of root tissue from two very closely related chickpea (*C. arietinum*) genotypes the landrace ICC 4958 and the popular local variety Annigeri, both considered to possess important sources of drought tolerance [35,36]. Following sequencing and functional annotation, we have generated over 100 EST markers which we have used in preliminary diversity analysis of wild and cultivated *Cicer *germplasm.

## Results and discussion

The SSH process resulted in over 4000 clones expected to be largely associated with ICC 4958 (SSH tester) but could conceivably also include some highly constitutively expressed genes from Annigeri (SSH driver). Typically the cDNA inserts ranged from 0.5–1 kb. All clones were partially or fully sequenced through a single pass read from the 3' end and trimmed of vector and low quality sequences. A length threshold of 170 bp was set in view of the minimum expected size of a functional gene-encoding region (exon) [37]. Removal of short sequences (below 170 bp: largely comprising of problematic repeats and sequences predominantly composed of poly-A) resulted in 2858 high quality EST sequences with an average length of 480 bp all available at the ICRISAT Chickpea EST Database [38].

### Analysis of ESTs and assembly of consensus sequences

**Figure 1:**
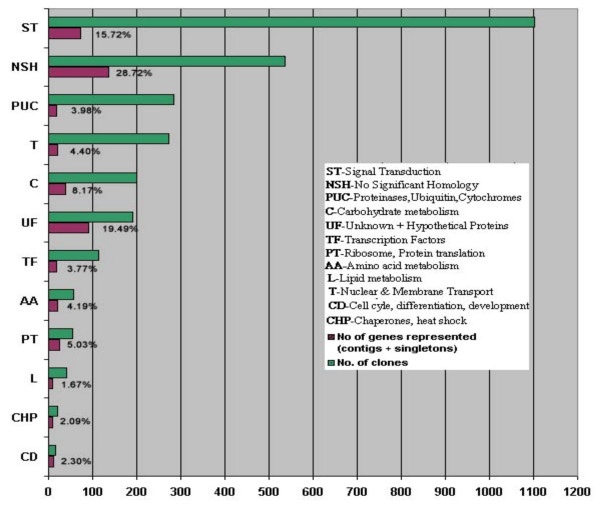
A summary of the number of EST clones and respective gene contigs classified in various functional categories based on alignments with public databases. Putative identifications were assigned to 2130 of the 2858 sequences generated from a chickpea root subtractive hybridization library. Percentages indicate the proportion of unigenes from the total number of unigenes identified in whole dataset.

Gene annotations were based on similarities to either known or putative ESTs in the public databases. All annotations were based on Blast searches, with a score threshold of ≥ 200 for BLASTn. For tBLASTx a score threshold of >100 was set, as these generally had e-values <10^-5 ^with a minimum of 50% identity over at least 30% of the length of the protein, which are the commonly used thresholds for reliable sequence annotation [39,40]. Tentative functional annotations of EST sequences based on tBLASTx were grouped under 12 general categories based on the biochemical functions of the predicted proteins (Figure 1). The 2858 sequences were assembled into 210 contigs with 267 ESTs remaining unassociated (singletons). Putative identifications could be assigned to 2179 of the 2858 sequences (76%), representing 73% of the unigenes (178 singletons and 162 contigs), and all of these proteins were represented in other plant species. The 2858 sequences were assembled into 210 contigs with 267 ESTs remaining unassociated (singletons). Assuming that in most cases each contig represents one gene [41] a maximum number of 477 genes are represented in this dataset (available at NCBI: accession numbers CK148643–CK149150). The total length of contigs ranged from 240 to 1291 bases (with an average of 648 bases) while the largest number of ESTs assembled into one contig was 255. EST assembly leading to the generation of tentative consensus (TC) sequences has dramatically reduced the redundancy in this database, as reported elsewhere [42] (see Additional file 1).

Over three quarters of the 210 consensus sequences (77% equivalent to 164 contigs) were found to have significant similarity (tBLASTx >100) to sequences in public databases. Many of these annotations (47 consensus sequences) were validated through comparison with the CDD database using RPS-BLAST which compares a protein query sequence to a position-specific score matrix prepared from the underlying conserved protein domain alignment [43]. Conserved motifs consistent with tBLASTx annotations were identified in 47 consensus sequences providing an additional level of confidence for these assigned gene functions (see Additional file 2).

As expected, we observed a substantial number of the EST contigs and singletons to have no significant homology (NSH: 48 contigs and 89 singletons; 29%) to either nucleotide or protein sequences in public databases at the time of analysis. This compares well to the higher proportion of sequences (44%) reported with little or no homology from chickpea leaf tissue [44]. In addition, a substantial number of ESTs (approximately 19% of the unigenes) were found to have similarity with unannotated hypothetical, putative or unknown proteins contained in the public databases and were, therefore, placed in the unidentified function (UF) class.

### PCR optimisation and unit costs

**Figure 2:**
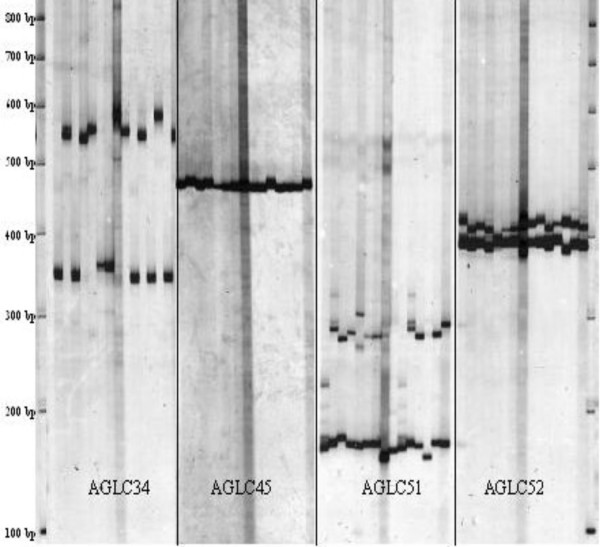
Polymorphic profiles of EST primers AGLC34, AGLC45, AGLC51 and AGLC52 screened on representatives of 8 wild species (IG69947, IG69960, IG69961, IG69974, IG69976, IG69986, IG69992, IG70029, ICC17116, ICC17121, ICC17122, ICC17126, ICC17141, ICC17148) and one cultivated genotype (ICC4958), separated by 6% non-denatured polyacrylamide gel electrophoresis and visualized by silver staining.

Optimization of PCR conditions is a critical precursor for accurate and robust large-scale marker screening and offers additional benefits for reducing the unit cost of genotyping [45]. As optimization relies on the sequential investigation of each reaction variable, the process can take a long time, thus the conditions of optimization are rarely identified empirically and individual components are rarely all tested simultaneously. However, Cobb and Clarkson [46] devised a strategy in which the components of a PCR reaction could be tested together using the least number ofexperiments based on the Taguchi principle [47]. We adapted the Cobb and Clarkson [46], 9 reactions of 4 PCR components to a 5 reactions of 5 PCR components, the additional component being enzyme concentration. This further reduced the time and cost of optimization, whilst also minimizing the cost of the PCR screening by also considering enzyme concentration. Final primer (0.15 - 0.5 pmol) and Taq DNA polymerase (0.2 – 0.5 U) concentrations resulted in a reagent unit cost of US$0.08 per PCR sample. This compares very favorable with other reports of PCR unit costs [48,49]. Clearly DNA extraction costs are an equally important component for molecular breeding programs and can also be greatly reduced as has been reported elsewhere [50]. The primer sequences and optimization parameters of all the polymorphic markers are given in Table 2. Optimization also resulted in fewer spurious amplification products and clearer polymorphic bands amongst the accessions studied (Figure 2).

### EST marker diversity analysis

A total of 106 EST primers were designed, optimized and screened, of which 48 gave good amplification products in ICC 4958 (the SSH tester accession in the EST generation process) while 58 were deemed unsuitable as they either did not produce an amplification product or generated a complex pattern of bands which were difficult to evaluate. Of the 48 primers pairs screened across 1 cultivated and 8 wild *Cicer *species, 44 were polymorphic (Table 2). A total of 167 polymorphic fragments ranging in size from 200 to >1000 bp was scored on polyacrylamide or agarose electrophoresis gels depending on product size (Figure 2). The number of alleles detected per primer ranged from 2 – 8 with the polymorphic information content (PIC) ranging 0.03 – 0.89 (Table 5).

Fourteen of the 106 EST's contained SSR motifs, of which 10 contained perfect trinucleotide repeats. A relatively large proportion of these EST-SSR markers (8) were derived from the no significant homology (NSH) and unknown protein functional (UF) classes, based on sequence comparisons (BLASTn tBLASTx and PSI-BLAST). Markers from these EST classes have readily detected polymorphism in the diverse germplasm tested (PIC 0.20–0.82). Since over 48% of the EST's generated from the chickpea root library fall into NSH and UF classes, these are clearly useful markers with potentially important, yet unknown functions.

Eleven of the polymorphic EST's were from stress annotated transcripts (AGLC2, AGLC16, AGLC 20, AGLC29, AGLC 34, AGLC45, AGLC52, AGLC53, AGLC55, AGLC66, AGLC68 and AGLC93); of these four contained SSR motifs (AGLC 34, AGLC55, AGLC67 and AGLC84). These stress related EST markers may be useful for allele-mining of germplasm collections for the identification of candidate accessions with new sources of agronomically important traits and for candidate gene mapping [51].

**Figure 3:**
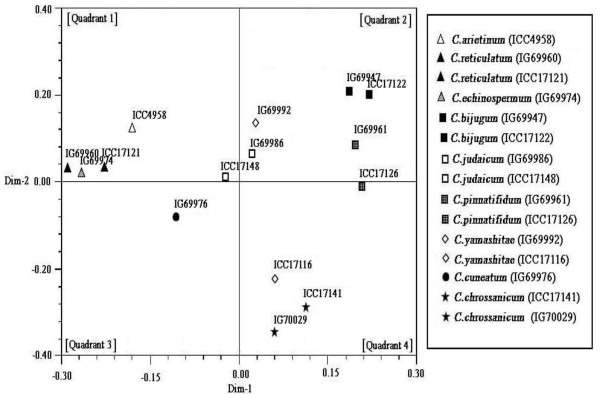
Principle Coordinate Analysis (PCoA) plot from diversity analysis of 44 EST markers screened across 14 accessions representing 8 wild *Cicer *species and one cultivated genotype (ICC4958).

**Figure 4:**
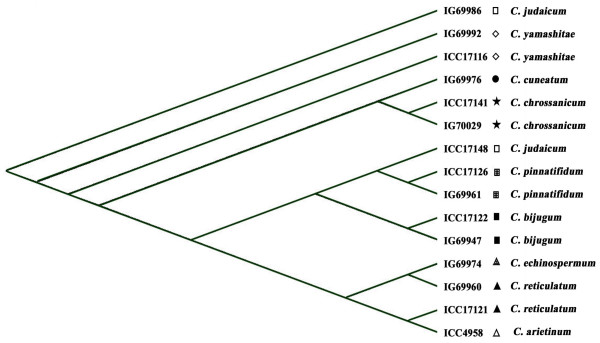
Dendogram of 14 *Cicer *accessions (representing 8 wild species and one cultivated genotype, ICC4958) based on parsimony analysis of 103 EST alleles.

The EST data-set generated in this study was used to compute pair-wise genetic distances between species according to Band similarity coefficient and UPGMA clustering. In order to display, with minimal distortion, the genetic relationships between species, a Principle Coordinate Analysis (PCoA) was carried out. The first two dimensions of the PCoA plot indicate the presence of 5 clusters, which account for 20% and 17% of the total variation (Figure 3). Dollo and polymorphism parsimony analysis (DOLLOP) conducted on this data-set found one most parsimonious tree with 301 steps (Figure 4) corresponding well with the PCoA analysis.

Both PCoA and parsimony analysis provide clear separation of most species. Both analysis grouped *C. echinospermum*, *C. reticulatum *and *C. arietinum*, together. These three species are classified together as the primary gene pool based on crossability studies. Similarly, all previous diversity studies based on allozymes [52], RAPD [16,53], microsatellite [54], ISSR [15,55] and AFLP [17] markers have also grouped *C. echinospermum*, *C. reticulatum *and *C. arietinum *together. The EST-based diversity analysis presented here shows *C. arietinum *(believed to be the wild annual progenitor [56] of cultivated chickpea) to be slightly distant to *C. echinospermum *and *C. reticulatum *but still within the same cluster (best reflected by the PCoA analysis Figure 3). All previous molecular diversity studies have clustered *C. bijugum*, *C. pinnatifidum *and *C. judaicum *together, although sometimes not well differentiated from the primary gene-pool species based on molecular diversity analysis. These three species are commonly considered to represent the secondary gene-pool as they require embryo rescue to recover viable progeny when crossed with the cultigen [6]. Both parsimony and PCoA analyses of the EST data-set clustered *C. judaicum*, *C. pinnatifidum *and *C. bijugum *together. This is in agreement with previous studies which demonstrated the close association of these three species. The current analysis suggests that *C. pinnatifidum *and *C. judaicum *are more closely related. This is in agreement with previous reports based on morphology analysis [56], seed storage protein analysis [57] and isozyme analysis [12]. In contrast, ecogeographical studies of the wild *Cicer *germplasm have suggested that *C. judaicum *to be quite diverged [7], which may explain the outlying position of one *C. judaicum *accession studied here (IG 69986) (Figure 4). Most other species generally fall into one of two clusters generally collectively referred to as the tertiary gene pool (Figure 3 and Figure 4) from which no viable hybrids have been reported from crosses with the cultigen.

Parsimony analysis grouped *C. chorassanicum *with *C. cuneatum*, but placed the *C. yamashitae *accessions as outliers (Figure 4). This data supports the tertiary group proposed by Croser et al. [6] to consist of; *C. chorassanicum *which has never been successfully crossed with cultivated chickpea and *C. yamashitae *and *C. cuneatum *which have been crossed with the cultigen but the resultant progeny have failed to flower or proven sterile. However, this is not reflected in the PCoA (Figure 3).

The distribution of accessions from the same species within secondary and tertiary clusters suggests that these two gene-pools may not be as distinct as previously expected. This may indicate that there are some tertiary gene-pool species that might be more readily crossed with the cultigen than their taxonomic classification would otherwise indicate. However, since the current study is based on a limited numbers of accessions for both *C. cuneatum *and *C. yamashitae *further research is required to fully define the relationships between these species and the scope of new opportunities for plant breeders.

## Conclusion

This is the first report of the use of EST-based markers for estimating genetic distances between annual *Cicer *species. We report on the development and characterization of 106 EST markers from chickpea sequences with functional annotations or unknown functions, some of which contain SSR motifs. These markers have detected high levels of polymorphism amongst the wild species studied here and initial results indicate that around 20% are polymorphic in intraspecific (*C. arietinum*) mapping populations, either directly or as cleaved amplified polymorphic sequences (CAPS) based markers [58].

The EST marker-based diversity analysis reported here broadly supports the *Cicer *taxonomy based on allozymic data and conclusions by Croser et al. [6]. The concurrence of our EST data with that of allozymic studies in particular suggests that many gene-based loci do have a common ancestory across *Cicer *species, as proposed but not substantiated by Choumane et al. [54]. Clustering patterns based on RAPD, AFLP and ISSR are generally very similar [15,17,55] but are substantially different to those based on the EST marker analysis reported here and the previously reported allozyme-based analyses [59]. This may be a consequence of RAPD, AFLP and ISSR markers sampling fundamentally different regions of the genome (under different evolutionary pressures) as compared with markers based on expressed genes. Unfortunately a detailed comparison of different assays is confounded by the virtual absence of common accessions and the adoption of different statistical analyses in each study [6].

The EST database developed in this study provides a preliminary profile of some differentially expressed genes that may be associated with constitutive mechanisms important for stress tolerance and root development in chickpea roots. These include transcripts with putative annotations for proteases, T6P synthase, non-specific lipid transfer proteins, MRP-like ABC transporters, chaperones- HSP70, TCP-1-alpha, bZIP transcription factor, calcium ATPases, protein kinases, MRP4 glutathione-conjugate transporter, glutathione S-transferase, phosphoenol pyruvate carboxylase, S-adenosyl methionine synthetase (see Additional files 1 and 2 for full details). It is envisaged that these differentially expressed genes can be validated by the chickpea community and the most promising ESTs used in candidate gene mapping and allele mining. The resultant annotated EST markers will then be of substantial value for marker-assisted introgression programs based on interspecific crosses using wild *Cicer *species that harbour agronomically valuable genes. One of the aims of chickpea breeding is to address the continuing need for cultivars adapted to particular geographical regions or with specific new ideotypes to ensure sustainability and profitability of production. An important means of continuing to achieve such breeding objectives is through the use of novel germplasm [60]. Many breeding programs, in particular those involving rice and wheat, have successfully utilized molecular and statistical approaches in accelerating the introduction of novel genes from wild species through marker accelerated backcross breeding.

The availability of increasing amounts of sequence data in many legume species now offers the potential for routine development of gene-based markers. These provide the ultimate assay (or so-called 'perfect marker') for indirect trait selection and map-based cloning. Combining gene-based markers together with highly polymorphic flanking SSR markers will greatly assist in reducing linkage drag and increasing the speed and efficiency of subsequent introgression programs. Finally, molecular genetic diversity analysis based on EST markers may also provide a means of predicting which accessions are most likely to produce fertile progeny when crossed with chickpea cultivars.

## Methods

### Plant material and growth conditions for SSH

Fifteen seeds from each chickpea accession (ICC 4958 and Annigeri) were sterilized with 25% chlorex (v/v) for 10 mins, rinsed twice with sterile distilled water, before placing in pre-sterilized plant pots containing sterilized vertisol. The soil was collected from the field, passed through a 2 mm sieve and autoclaved in plastic bags using a 60 min cycle at 120°C and repeated three times on consecutive days. Seeds were sown immediately after the last autoclave cycle.

Three seeds were sown per pot; the plants were maintained in a Conviron growth chamber (Conviron, Winnipeg, Man.) under optimal physiological conditions for chickpea growth: 25°C day (11 h) and 12°C night (13 h), 455 μmoles m^-2 ^sec^-1 ^illumination intensity, 80% day and 40% night relative humidity, and irrigation as required.

Roots were harvested 28 days after germination (with flowering expected after 30 days). The soil was washed off the roots in running water, the intact whole plant was lifted out of the soil and soil particles were gently rubbed off in running water with gloved hands. The root tissue was subsequently treated with (0.1% v/v) DEPC solution and immersed in sterile Milli Q water, excess water from the roots was blotted onto filter paper and roots were weighed (approx. 2 g), flash frozen in liquid nitrogen and stored at -80°C.

### RNA isolation and suppressed subtractive hybridization

The subtraction of the two genomes was achieved through a process of PCR-based suppression of genes common to both genotypes, and genes that are differentially expressed are enriched. As the process is based on PCR, low copy number cDNA can be detected from the tester (ICC 4958). RNA isolation and library construction was carried out by Avestha Gengraine Technologies (Bangalore, India). Total RNA was isolated from the root tissue of ICC 4958 and Annigeri using Trizol reagents (GIBCO-BRL) for RNA isolation, mRNA was purified using Oligotex (Qiagen GmbH, Hilden, Germany).

The suppression subtractive hybridization (SSH) process was carried out using a Clontech PCR-Select cDNA subtraction kit (Clontech INC. USA). Tester (ICC 4958) and driver (Annigeri) cDNA was restricted with *Rsa*I for 90 mins and cDNA from the ICC 4958 was ligated with the required adaptors. Adaptor ligation was confirmed by PCR, followed by one round of over-night hybridisation with Annigeri as the driver and ICC 4958 as the tester. A second round of hybridisation was followed by primary PCR (27 cycles) and secondary PCR (12 cycles) according to the manufacturer's instructions for the PCR-Select. The PCR products of the subtraction were analyzed by gel electrophoresis and cloned using pCR4-TOPO cloning kit (Invitrogen Inc., USA). The cDNA clones were amplified in *E. coli *DH10B cells by electroporation and insert containing clones were selected by plating on LB agar containing 100 ug/ml ampicillin. Large colony forms were picked and used to regenerate single clone cultures in 96-well microtitre plates. After growth over-night at 37°C, glycerol was added to a final concentration of 15% and cultures were stored at -80°C.

### EST sequencing

Cultures of transformed *E. coli *were grown over-night in 100 ul LB media containing 100 ug/ml ampicillin, the cells were centrifuged and suspended in sterile distilled water, heat denatured for 10 mins at 95°C, 1 ul of supernatant was used for insert amplification with M13 forward and reverse primers. Inserts (5 ul) were separated by electrophoresis on 1.2% agarose gel containing ethidium bromide to confirm amplification of a single insert and for quantification. After SAP/exonuclease I digest, 4 ul of the insert was sequenced using T_7 _primer and 1/8 fraction of the recommended volume of dye terminator reagent (Big Dye terminator cycle sequencing kit v2, ABI-Foster City) for cycle sequencing. After removal of dye blobs by ethanol precipitation, DNA sequences were analyzed by capillary-electrophoresis using an Applied Biosystems genetic analyzer model ABI 3700 or ABI 3100.

### Sequence analysis for predicting gene function

Base calling was performed using the ABI DNA Sequence Analysis Software (v3.7) or by Chromas v2.2 (Technelysium Pty Ltd. Australia). All sequences were scanned visually for quality of the peak shapes and corresponding base call. Vector sequences were trimmed using Sequencher v.4 (Gene Codes, Ann Arbor, MI) software and low quality bases (quality score <20) were trimmed from both ends of sequences. A total of 4000 sequences were analyzed, only sequences of more than 170 bp with less than 5% ambiguity were processed. All sequences containing interspersed or simple repeats were masked with the RepeatMasker software [61] using *Arabidoposis *as reference. The masked sequences were screened against the following DNA databases: *Arabidopsis thaliana*, *Medicago truncatula*, *Glycine max*, *Zea mays *and *Oryza sativa*, in the TIGR Gene Indices using default settings for BLASTn and tBLASTx searches [62]. The same sequences were also used to search the NCBI EST database (dbEST) using *Viridiplantae *as a limiter [63].

The comparison of chickpea sequences with those in public databases was carried out during 2002 – 2003, and blast searches with unique sequences were repeated in November 2004. An in-house script was used to retrieve best matches from the outputs of BLASTn and tBLASTx text files, retaining the following information: gene identifier, description, BLAST score, percent identity, alignment length, reading frame, and database name from the top-scoring alignments. Alignments with scores of ≥200 for BLASTn and ≥100 for tBLASTx were extracted into an MS-Access table. The individual ESTs were assembled into tentative consensus sequences (TCs) using Sequencher, the assembly parameters used for 'dirty data' were (minimum percentage match of 90 and minimum overlap 40 bp). A sequence was retained in a contig if it matched at least one sequence already in the contig based on pair-wise BLAST [63], with a percentage identity threshold of 90%. A consensus sequence was then derived for each cluster. Consensus sequences were translated into six possible reading frames with Transeq software [64], using the standard genetic code. The translated sequences were then submitted to Reverse Position Specific (RPS)-BLAST [63] to search for matches to the conserved domain database (CDD; all 4540-PSSMs) using default parameters. The RPS-BLAST outputs were similarly processed using an in-house script, retaining the following information from the top-scoring alignments: gene identifier, description, score and e-value. Each result was manually interpreted and classified according to the biochemical function of the predicted protein.

### Plant material and DNA isolation

Fifteen chickpea accessions representing 9 annual *Cicer *species, including the cultivated species *C. arietinum*, were used in this study (Table 1). Seeds of these accessions were germinated in Jiffy pots in a growth room and DNA was extracted from 4–5 pinnules of 15–20 day old individual plants with 2% CTAB extraction buffer containing 0.03% mercaptoethanol as described in [50]. DNA concentrations were determined by comparing the sample intensity (1 μl) with that of known amounts of uncut lambda DNA by electrophoresis in 1.2% 'Ready-to-run' agarose gels (Amersham) containing ethidium bromide. DNA was diluted to a working stock concentration of 10 ng/μl and checked before use.

### Primer design of EST markers

A total of 106 EST sequences were chosen that represented diverse functional/stress annotations, a selection of which contained SSR motifs. In addition, primers were also designed from a random selection of sequences categorized as having 'no significant homology' (NSH) and 'unknown protein function' (UF) when compared with either nucleotide or protein sequences in public databases.

Primer sequences were designed using Genefisher 1.1 software [65] using the following criteria 57 – 63°C melting temperature (Tm), 40–50% GC content, 17–24 bp primer length and the difference in Tm between forward and reverse primer pairs was limited to 2°C. For those EST sequences containing repeat motifs, primers were designed to flank the microsatellite region. Optimal primer pairs were selected using Netprimer, the primer design algorithm which analyzes all possible secondary structures including hairpins, self-dimers and cross-dimers [66]. The Tm and product length of 8 forward and reverse pairs per sequence from Genefisher software were evaluated by Netprimer software for minimal or no secondary structure. All primer and EST sequences are accessible through the ICRISAT Chickpea EST Database [38].

### PCR optimization and evaluation

The chickpea accession ICC 4958 (from which the EST sequences were derived) was used as the reference accession for primer optimization. A total of 106 EST primers (synthesized by MWG, Germany) were optimized simultaneously for the following PCR components using a modified (5×5) grid [46]. On this basis initial optimization of the five major components in a PCR (concentrations of primer, template DNA, Mg^++^, dNTP and enzyme) were empirically determined for each primer used in this study (Table 3). Optimal touch-down temperature and number of amplification cycle profiles were also determined for each primer pair (Table 4). This procedure is important to minimize non-specific amplification, maximize data accuracy and minimizes cost.

PCRs were performed in a total volume of 5 μl containing 5–10 ng of DNA and optimized concentration of the following: primer (0.15–0.3 pmoles), dNTP (0.1–0.2 mM), MgCl_2 _(1.5–2 mM), Qiagen Taq (0.2–0.5 u) and 1 x buffer. PCR amplifications were carried out using a GeneAmp model 9700 thermocycler (Perkin Elmer-Applied Biosystems). As the annealing temperatures of the markers ranged from 47–65°C we opted to use 3 catagories of "touchdown" temperature cycles (see Table 4).

PCR amplified products with a size range of 200–600 bp were resolved by non-denaturing polyacrylamide gels electrophoresis (6–10% acrylamide/bisacrylamide, 20:1 in TBE). Bands were visualized through a modified silver staining protocol [67] as follows: gels were immersed in water for 3 mins, followed by 20 mins in 0.1% CTAB solution and 0.3% ammonia solution for 15 mins. Silver staining solution was freshly prepared each day, consisting of 0.1 % (w/v) AgNO_3 _in 4 mM NaOH solution, 0.5 to 0.6 ml of 25% ammonia was titrated until the cloudy suspension became clear. Gels were gently agitated in the silver nitrate solution for 30 mins, and developed in 1.5% (w/v) sodium carbonate and 0.02% (v/v) formamide solution until bands appeared. The gels were rinsed in water, fixed in 1.5% glycerol solution and documented by scanning. PCR products above 600 bp were resolved by agarose gels electrophoresis (0.8–1%) containing ethidium bromide.

### EST marker data collection and analysis

The amplification profile from each EST marker was checked for reproducibility and subsequently scored visually (and independently rescored) for the presence (1) or absence (0) of polymorphic bands. The degree of polymorphism was quantified using the polymorphic information content (PIC) calculator [68] based on the following formula:

PIC = 1 - ∑ *Pi*^2 ^- ∑ ∑ *Pi*^2^*Pj*^2^

i = 1 i = 1 j = i+1

where *Pi *is the frequency of an individual genotype.

Pair-wise genetic distances were calculated with NTSYS-pc software version 2.0 [69] as S_xy _= 1 - (2n_xy_/n_x _+ n_y_) as S_xy _= 1 - (2n_xy_/n_x _+ n_y_) based on band coefficient [70], where n_xy _are shared bands amongst the individuals, n_x _and n_y _are the number of fragments exhibited by each individual. Distance based on Band coefficient is similar to the genetic distance (GD) formula by Nei and Li [71]: GD = 1 - S_xy_. The 15 × 15 similarity matrix (based on Band coefficient) was subjected to sequential agglomerative hierarchical nested (SAHN) clustering using UPGMA (unweighted pair-group method analysis). Principle coordinate analysis based on these distance estimates was performed using NTSYS, using consecutive commands of 'DCENTRE' and 'EIGEN' in order to generate a PCoA plot which is more informative in highlighting differences between major taxonomic groups than dendrogram representations. Maximum parsimony analysis was conducted using the PHYLIP program Dollo and polymorphism parsimony analysis version 3.63 [72] and the consensus tree viewed in tree view.

## Authors' contributions

HKB coordinated library construction and sequencing of a large proportion of the ESTs, experimental design, marker analysis and manuscript development. JB coordinated and/or carried out all bioinformatic analyses and participated in all aspects of manuscript development. KE carried out all aspects of marker optimization and screening, and contributed to various aspects of experimental design and data analysis. JHC participated in all aspects of experimental design, data interpretation and manuscript development and provided overall coordination of the project. All authors read and approved all versions of the manuscript.

## Supplementary Material

Additional File 1 **List of the most abundant EST from the chickpea root EST database. **Number of chickpea ESTs forming tentative consensus sequences (TC) with corresponding TC number and where known, putative annotation*. Click here for file

Additional File 2 **Table of chickpea unigene sequences classified through RPS-Blast. **Functional categories, with corresponding genbank ID numbers, Blastx descriptors, domains identified through RPS-Blast and e-values of chickpea unigene sequences. Click here for file

## Figures and Tables

**Table 1:** Passport data of *Cicer *accessions used in this study.

**Accessions**	**Species**	**Alternate accession identifier**	**Biological status**	**Origin and location**	**Latitude**	**Longitude**
ICC4958	*C. arietinum*	JGC 1	Cultivar	India	no data	no data
IG69947	*C. bijugum*		Wild	Turkey (Diyarbakir)	40.0	33.1
ICC17122	*C. bijugum*	ICCW 7	Wild	Turkey (Savur)	37.5	40.9
IG70029	*C. chorassanicum*		Wild	Afghanistan (Bamian)	67.5	34.5
ICC17141	*C. chorassanicum*	ICCW 26	Wild	Afghanistan (Shahidan)	34.2	69.7
IG69976	*C. cuneatum*		Wild	Ethiopia (Tigray)	38.5	14.1
IG69974	*C. echinospermum*		Wild	Turkey (Sanli Urfa)	30.4	37.4
IG69986	*C. judaicum*		Wild	Syria (Tartous)	35.5	35.0
ICC17148	*C. judaicum*	ICCW 33	Wild	Lebanon	no data	no data
IG69961	*C. pinnatifidum*		Wild	Turkey (Elazig)	39.2	38.4
ICC17126	*C. pinnatifidum*	ICCW 11	Wild	Turkey	38.7	39.3
IG69960	*C. reticulatum*		Wild	Turkey (Mardin)	40.6	37.3
ICC17121	*C. reticulatum*	ICCW 6	Wild	Turkey (Savur)	37.5	40.9
IG69992	*C. yamashitae*		Wild	Afghanistan (Kabul)	69.4	34.4
ICC17116	*C. yamashitae*	ICCW 1	Wild	Afghanistan (Shezghan)	34.7	69.7

**Table 2:** Characteristics of chickpea EST markers developed from EST root library with primer sequence, GenBank accession numbers, PCR optimized parameters including touch down amplification profile.

**Marker**	**Primer Sequence (5'--3') Forward**	**Primer Sequence (5'--3') Reverse**	**GenBank accession numbers**	**Touch -down profile (°C)**	**PCR optimised reagents**			
					**Primer (pmol)**	**Mg++ (mM)**	**dNTP (mM)**	**Taq (u)**
**AGLC2**	TGTCAGACTGAGCTGTGTATGAGA	TTGCCCGTATGGTTATGTTAGGAA	CK148953*	55–45	0.3	1.5	0.15	0.3
**AGLC7**	GACCCCCAAAAATGAAAAAGCA	TTGCCCATACATTCTTCACCCAA	CK148966	60–55	0.3	1.5	0.15	0.3
**AGLC8**	CAAACTCCTCAATAGCAGGCACA	GCTGTATCGGAGAGTGGTCAGA	CK149041*	55–45	0.3	1.5	0.15	0.3
**AGLC9**	ACTCCTGTAGTGGCATATCTTCGA	TGGTCCATTTATGCCGCTGGTA	CK148709	60–55	0.3	1.5	0.15	0.3
**AGLC14**	GCAGCAACTATTTACACTGGTA	CTCTCTGGGAGAAAGCTCGGAA	CK149086*	60-55	0.45	1.5	0.15	0.3
**AGLC15**	ACTGATCAAGGTCTCTTCTAGACA	CCCAACAAACTGGACAAAGCAGA	CK149086*	60-55	0.45	1.5	0.15	0.3
**AGLC16**	GAGTACTTGCCAACTAGCTTAGGA	TTGGATATAACAGATGACGGGGAA	CK149087*	60-55	0.3	1.5	0.15	0.3
**AGLC19**	GCATCCTTCCCACTTCTTTGCA	GAATGGACTCGGATGTCTTAAGCA	CK148924*	60-55	0.3	1.5	0.15	0.3
**AGLC20**	AATGGTGATTCGTCAGTCGCCTA	CTGTCTGAAGAAAGTGAACGAA	CK148978*	60-55	0.3	1.5	0.15	0.3
**AGLC27**	CAAATTTCTGTTCTTCCACCCCAA	GGCGATCTTCGAGTCCATCGA	CK148933	60-55	0.3	1.5	0.15	0.3
**AGLC28**	GCTAAACCTTAGAGCAATGACTCA	CCTTGCTTGTGCCTTATCTTCCA	CK148934	60-55	0.3	1.5	0.15	0.3
**AGLC29**	TCTTCAACACCTCCATCTAACCTA	GACATGAAACCAAAGCATCACA	CK148945*	60-55	0.3	1.5	0.15	0.3
**AGLC34**	CTTTACCAAAACCACCTTCACCAA	TCTCTCTCTCTCTCTTCTGTTCCA	CK149140*	60-55	0.5	1.5	0.15	0.3
**AGLC39**	GCCGAGGTACACTTTACCAA	TCCTCACACTTCAGGTTCAACGA	CK149136	55-45	0.5	1.5	0.15	0.3
**AGLC45**	CTCCTCTTCTCCGTCGTAGCA	CTGGTCCTTGACGGGAGTGA	CK149070*	65-60	0.5	1.5	0.15	0.3
**AGLC47**	GTTTACATCATGACCCGCCCTA	TCACCAAGACCAGAACGTTCCA	CK148819	60-55	0.5	1.5	0.15	0.3
**AGLC48**	TGCCCAACGGTTTCTTTTACCA	TCAGAGATACTCGCCCACCAA	CK148960	55-45	0.5	1.5	0.15	0.3
**AGLC51**	TCTTTGAGCAGCATTCATTCCACA	GAGTGCTACCTTCAAAGACTGCA	CK148677	55-45	0.5	1.5	0.15	0.3
**AGLC52**	CGATCAAGAACCCAGTTTTGCAA	AAGATCGACAGGCGATCTGGTA	CK148718	55-45	0.5	1.5	0.15	0.3
**AGLC53**	CACTCTCCGTTCCGGTTCCA	CTGTCCATGCCCTTGTCCA	CK148806	60-55	0.3	1.5	0.2	0.5
**AGLC55**	CAGGTCGCGTTGTTGCA	GGCCGAGGTACACTTTTCCA	CK149133*	60-55	0.5	2	0.1	0.3
**AGLC57**	TTCATCTGGCACTAGCATATCTGA	CGACAATTCTTGCTTCAACAACCA	CK148900*	60-55	0.3	2	0.1	0.3
**AGLC60**	CATGTTTTCTACCCTCACAATGCA	TACTCACTTGTTGTTCCAGACA	CK149124*	55-45	0.2	1	0.1	0.2
**AGLC61**	TTCGATCCTCCGACCCCGAA	TTCGCTAGATCTGGATACTTCTCA	CK148802	60-55	0.3	2	0.1	0.3
**AGLC64**	TCTTCTTCTTCTTCTTCAGCCACA	GTGGATTGGGAAATGTGAATGTCA	CK149128*	55-45	0.2	1	0.1	0.2
**AGLC66**	CCACAAAGGACGACAACAACGA	CCCAACACGAACCACACGA	CK149070	60-55	0.2	2	0.2	0.5
**AGLC67**	ATCCATCACAACCCTCAACTCA	CTCCGTCAACCTTTCCGCAA	CK149102*	55-45	0.3	2	0.1	0.3
**AGLC68**	TGTTGTCTCGCCAATTCAAAGCA	CGTTTGGTGGCATTCCTGCA	CK148906*	55-45	0.3	2	0.1	0.3
**AGLC72**	TTTAATTACGCGGTTTCCACGA	GAAGACTTGAGACATGGGCACA	CK148928*	55-45	0.2	1	0.1	0.2
**AGLC74**	CGTGGGATTGAAAAAGTTGCTA	CACTACCAGCCAAAGCACTCA	CK149006*	55-45	0.2	1	0.1	0.2
**AGLC75**	CAACAACAACCTATCCGAACCTCA	ACTATCCCTAACCTTCCATCACCA	CK148853	60-55	0.5	1	0.2	0.5
**AGLC76**	CATGAGTGGTAGTGGGAGTGGA	GTTCGTTTGAGTCGTTTACTGGAA	CK148862	60-55	0.2	2	0.2	0.5
**AGLC78**	TCAACAACGCTACCCGATCCAA	TTCTCAAGAGCACCACAAAAGAGA	CK149132	60-55	0.3	2	0.1	0.3
**AGLC79**	CGGCGGCTATATTGGTTTTGCA	TCCTAAACCCCACTTATCTCCCTA	CK149080*	60-55	0.3	2	0.1	0.3
**AGLC82**	TTTGTGATGGTCCTGCTCTCTCA	ACCGCTTCAGGATCAACTCGA	CK148871	60-55	0.3	2	0.1	0.3
**AGLC83**	TCTTCCGATCCTAAGAAAGAGCAA	ACCAATATGGAGAGCACCAGTCA	CK148894	60-55	0.3	2	0.1	0.3
**AGLC84**	CCACCTTCCATCTCCAATTCCAA	GACTGAATCGGAGAAGGTTTCTCA	CK149089	60-55	0.15	2	0.1	0.2
**AGLC85**	CCAGCTTCTAATGTAGGTCTGCA	CAGCAGCAGCAGAGAGAGCA	CK148761	60-55	0.15	1	0.1	0.2
**AGLC88**	ACTTGGGCGTTCAAAAATCTCA	CCATTACGATCAAAGAGCTCAGGA	CK148768	55-45	0.3	2	0.1	0.3
**AGLC93**	GTCCGAGCTGTGGATAGGGAA	GTTCCGCCTTCAATCCATGGAA	CK148904*	60-55	0.2	2	0.2	0.5
**AGLC94**	CCAACTTCCCTCATTCTTATTCCA	ACCAATTCCAAATTTCCAGCTCGA	CK149093	55-45	0.2	1	0.1	0.2
**AGLC96**	TCCATATGGCTGAAGAACCCCAA	TTCTGAGGTTCAGGTAGTTCGGAA	CK149016*	60-55	0.2	2	0.2	0.5
**AGLC98**	CTCTTTCTTTCCCTCTAGTTTCCA	CGGCGAACTCGTGTTTGCTA	CK149016	55-45	0.2	1	0.1	0.2
**AGLC101**	TGTCCAAAATTGGGATCAGAGA	AGAACGACTTCAGCAGCAGCA	CK148993*	55-45	0.3	2	0.1	0.3

*Genbank accession numbers of the longest EST sequence of the contig, used in primer design.

**Table 3:** Permutations of PCR component concentrations used for optimization of PCR conditions for each marker.

**Reactions**	**Primer (pmol)**	**Template (ng)**	**MgCl**_2 _**(mM)**	**dNTP (mM)**	**Enzyme (u)**
1	0.2	5	1	0.1	0.2
2	0.2	15	2	1.5	0.5
3	0.3	5	1.5	0.2	0.5
4	0.3	10	2	0.1	0.3
5	0.5	10	1	0.2	0.3

**Table 4:** PCR amplification profiles used for optimization of PCR conditions for each marker.

**Name**	**Profile**	**Temperature°C**	**Time min. sec**	**Number of cycles**
65-60	Denaturation	95	3.00	1
	Touch down	94	0.20	5
		65-60	0.20	
		72	0.30	
	Normal	94	0.20	30
		59	0.20	
		72	0.30	
	Extension	72	20.0	1
				
60-55	Denaturation	95	3.00	1
	Touch down	94	0.20	5
		60-55	0.20	
		72	0.30	
	Normal	94	0.20	30
		56	0.20	
		72	0.30	
	Extension	72	20.0	1
				
55-45	Denaturation	95	3.00	1
	Touch down	94	0.20	10
		55-45	0.20	
		72	0.30	
	Normal	94	0.20	30
		48	0.20	
		72	0.30	
	Extension	72	20.0	1

**Table 5:** Characteristics of chickpea EST markers including SSR motif where present, annotation information (NSH indicates no significant homology), and, size range of amplification products, number of alleles and PIC (polymorphism information content) values based on screening of germplasm in Table 1.

**Marker**	**SSR motif**	**Annotation**	**Product size (bp)**	**No. of alleles**	**PIC**
AGLC2		Valyl tRNA synthetase	620	2	0.50
AGLC7		D-3-phosphoglycerate dehydrogenase	330–335	2	0.44
AGLC8		RNA helicase	525–550	5	0.78
AGLC9		Unknown protein	680	4	0.62
AGLC14		Hypothetical protein	520–600	2	0.45
AGLC15		Hypothetical protein	520–600	4	0.65
AGLC16		WD-repeat protein like	365–370	2	0.32
AGLC19		Formin-like protein	350–355	4	0.50
AGLC20		Type IIB calcium ATPase	230–235	3	0.61
AGLC27		Putative Scarecrow gene regulator	330–335	3	0.43
AGLC28		Type 2A protein phosphatase	335–350	3	0.86
AGLC29		Non-specific lipid transfer protein	325–400	6	0.64
AGLC34	AG(20)	Putative protein kinase	350–600	5	0.75
AGLC39	AG(17)	No significant homology	275–290	3	0.74
AGLC45		Probable cysteine proteinase (EC 3.4.22.-)	410–420	2	0.29
AGLC47		(NM_127184) putative beta-ketoacyl-CoA synthase	200–210	2	0.59
AGLC48		Putative peroxisome assembly factor-2 (NM_100181)	400–410	3	0.89
AGLC51		RNA binding protein	170–180	4	0.55
AGLC52		Protein kinase	390–410	6	0.89
AGLC53		Acyl- [acyl-carrier protein] desaturase	390–400	5	0.87
AGLC55	CT(17)	Putative protein kinase	225–275	4	0.89
AGLC57	GGA(3)	Unknown protein; protein	>1000	3	0.56
AGLC60	TTC(3)	Hypothetical protein	310–350	7	0.82
AGLC61	GAA(3)	Protein of unknown function	450–470	5	0.55
AGLC64	TTC(6)	No significant homolgy	270–360	4	0.20
AGLC66		Probable cysteine protenase (EC 3.4.22.-)	500–650	8	0.63
AGLC67	TCT(4)	Tryptophan synthase beta-subunit (TSB2)	>1000	4	0.56
AGLC68		Lon protease homolog 1 precursor	220–230	3	0.39
AGLC72		Putative Grr1 protein	230–250	4	0.62
AGLC74	TTTA(3)	Glycosyl hydrolase family 17	400–450	5	0.75
AGLC75	AAC(5)	No significant homology	200–230	3	0.48
AGLC76	AAG(3)	RGA2 protein *Arabidopsis thaliana*	440–480	3	0.71
AGLC78	CAA(5)	Translation initiation factor eIF3 – like protein	430–450	2	0.44
AGLC79		No significant homology	330–410	5	0.03
AGLC82		Unknown protein; protein id: At1g80280.1	300–320	3	0.50
AGLC83		Putative valyl tRNA synthetase [*Oryza sativa*-japonica cultivar-group]	>1000	3	0.74
AGLC84	AAG(3)	DEAD/DEAH box RNA helicase	430–450	3	0.42
AGLC85		Emb trypsin inhibitor cme precursor	410–450	3	0.47
AGLC88		Chromomethylase CMT2 *Arabidopsis thaliana*	>1000	5	0.78
AGLC93		BZIP family transcription factor	>1000	2	0.51
AGLC94		T48 ankyrin-like protein	480–500	4	0.67
AGLC96		Hypothetical protein CAB95829.1	310–360	5	0.59
AGLC98	AG(19)	Hypothetical protein	405–450	5	0.78
AGLC101		No Significant homology	370–400	4	0.70
