# Supplementary material for: Development of ESTs from chickpea roots and their use in diversity analysis of the Cicer genus
Source: BMC Plant Biol. 2005 Aug 17;5:16. doi: 10.1186/1471-2229-5-16 (PMC1215491; doi:10.1186/1471-2229-5-16)
Supplement: Additional File 1 — List of the most abundant EST from the chickpea root EST database. Number of chickpea ESTs forming tentative consensus sequences (TC) with corresponding TC number and where known, putative annotation*. [file 1471-2229-5-16-S1.doc]

# Additional File 1: List of the most abundant EST from chickpea root EST database. Number of chickpea ESTs forming tentative consensus sequences (TC) with corresponding TC number and where known, putative annotation*.

| **TC sequence #** | **Number of ESTs in TC** | **Tentative annotation** |
| --- | --- | --- |
| TC 9 | 255 | Putative protein kinase |
| TC 23 | 152 | Cytochrome c oxidase subunit |
| TC 121 | 128 | Putative protein kinase |
| TC 108 | 128 | No significant homology/no putative annotation |
| TC 39 | 95 | WD-repeat protein-like |
| TC 24 | 63 | Putative O-linked GlcNAc transferase, At3g04240.1 |
| TC 30 | 55 | Putative protein kinase |
| TC 56 | 55 | Putative cytochrome oxidase Vc subunit, At2g47380.1 |
| TC 57 | 50 | Putative O-linked GlcNAc transferase, At3g04240.1 |
| TC 29 | 32 | Phosphate induced protein-1 |
| TC 26 | 20 | Non specific lipid transfer protein |
| TC 71 | 20 | WD-repeat protein like |
| TC 62 | 19 | Phosphate induced protein-1 |
| TC 51 | 18 | Arm repeat containing protein |
| TC 17 | 17 | Arm repeat containing protein |
| TC 34 | 16 | Transcription factor scarecrow |
| TC 63 | 16 | Protein kinase |
| TC 27 | 14 | Putative methylmalonate semi-aldehyde |
| TC 187 | 13 | Very long chain fatty acid condensing enzyme CUT1, At1g19440.1 |

* NSH/UF contigs with less than 100 ESTs have not been listed.
