# Supplementary material for: Development of ESTs from chickpea roots and their use in diversity analysis of the Cicer genus
Source: BMC Plant Biol. 2005 Aug 17;5:16. doi: 10.1186/1471-2229-5-16 (PMC1215491; doi:10.1186/1471-2229-5-16)
Supplement: Additional File 2 — Table of chickpea unigene sequences classified through RPS-Blast. Functional categories, with corresponding genbank ID numbers, Blastx descriptors, domains identified through RPS-Blast and e-values of chickpea unigene sequences. [file 1471-2229-5-16-S2.doc]

**Additional File 2: Table of chickpea unigene sequences classified through RPS-Blast.** Functional categories, with corresponding genbank ID numbers, Blastx descriptors, domains identified through RPS-Blast and e-values of chickpea unigene sequences.

| Functional category | Genbank ID* | No. of ESTs | Nr ID. | Blastx description | Domain annotation from CDD | E-value (from RPS-Blast) |
| --- | --- | --- | --- | --- | --- | --- |
| **Signal transduction** | TC18 (CK148909) | 9 | T51247 | ARR2 protein | Response regulator containing CheY-like receiver, AAA-type ATPase, and DNA-binding domains | 1.00E-21 |
| **Signal transduction** | TC83 (CK148959) | 4 | T51247 | ARR2 protein | REC, cheY-homologous receiver domain | 1.00E-15 |
| **Signal transduction** | TC84 (CK148929) | 4 | AAM83219.1 | At4g03080 | PP2Ac, Protein phosphatase 2A homologues, catalytic domain | 1.00E-18 |
| **Signal transduction** | TC204  (CK149090) | 2 | AAK92639.1 | Putative protein phosphatase | pfam02383, Syja_N, SacI homology domain | 1.00E-43 |
| **Signal transduction** | CK148837 | Singleton | NM_129310 | Putative ADP ribosylation factor1 GTPase | Arfgap, putative GTPase activating protein | 2E-22 |
| **Signal transduction** | CK148651 | Singleton | AJ292981 | SRPK4 | Serine/threonine kinases, catalytic domain | 8E-4 |
| **Signal transduction** | CK148702 | Singleton | AAM65362 | Asp1 | ArfGap, putative GTPase activating proteins for the small GTPase ARF | 4E-25 |
| **Signal transduction** | CK148870 | Singleton | AAD46038 | Nucleic acid binding protein | RRM, RNA recognition motif | 6E-10 |
| **Unknown protein** | TC19 (CK149024) | 7 | NP_566201.1 | Expressed protein; protein id: At3g03310.1 | LACT, Lecithin:cholesterol acyltransferase. | 8.00E-04 |
| **Unknown protein** | TC172 (CK149036) | 2 | NP_172250.1 | Hypothetical protein | ANK, ankyrin repeats | 4.00E-18 |
| **Unknown protein** | TC175 (CK148912) | 3 | NP_176723.1 | Unknown protein | YqgFc, Likely ribonuclease with RNase H fold | 3.00E-11 |
| **Unknown protein** | TC52 (CK149034) | 6 | T51247 | ARR2 protein | REC, cheY-homologous receiver domain | 9.00E-17 |
| **Unknown protein** | TC179 (CK14890) | 3 | NP_189908.2 | Putative protein; protein id: At3g43220.1 | pfam02383, Syja_N, SacI homology domain | 1.00E-06 |
| **Unknown protein** | TC210 (CK149029) | 2 | AAM63377.1 | Putative UPF0183 protein | Uncharacterized protein family (UPF0183) | 6.00E-18 |
| **Carbohydrate metabolism** | TC22 (CK148965) | 8 | NP_186955.1 | Putative cellulose synthase catalytic subunit | pfam03552, Cellulose synthase. | 4.00E-108 |
| **Carbohydrate metabolism** | TC24 (CK148979) | 64 | NP_187074.1 | Putative O-linked GlcNAc transferase | Spy, Predicted O-linked N-acetylglucosaminetransferase, SPINDLY family | 3.00E-39 |
| **Carbohydrate metabolism** | TC55 (CK149050) | 4 | AAO03579.1 | Cellulose synthase-like protein D4 | Cellulose synthase | 1.00E-101 |
| **Carbohydrate metabolism** | TC57 (CK149092) | 51 | NP_187074.1 | Putative O-linked GlcNAc transferase | Spy, Predicted O-linked N-acetylglucosaminetransferase | 1.00E-38 |
| **Carbohydrate metabolism** | TC85 (CK148907) | 2 | NP_197193.1 | Cellulose synthase catalytic subunit -like | Cellulose synthase. | 0.002 |
| **Carbohydrate metabolism** | TC87 (CK149017) | 10 | NP_187074.1 | Putative O-linked GlcNAc transferase | Spy, Predicted O-linked N-acetylglucosaminetransferase | 2.00E-35 |
| **Carbohydrate metabolism** | TC174 (CK149009) | 4 | NP_176656.1 | Glycosyl hydrolase family 17 | pfam00332, Glyco_hydro_17, Glycosyl hydrolases family17 | 4.00E-24 |
| **Carbohydrate metabolism** | TC194 (CK148899) | 2 | BAB55475.1 | Putative fructose-bisphosphate aldolase | Fructose-bisphosphate aldolase class-I | 1.00E-11 |
| **Carbohydrate metabolism** | TC201 (CK149099) | 2 | AAC67586.1 | Pyrophosphate-dependent phosphofructokinase | Pyrophosphate_PFK, Phosphofructokinase | 3.00E-68 |
| **Carbohydrate metabolism** | TC202 (CK149073) | 2 | AAF85975.1 | 3-phosphoglycerate kinase | Pgk, 3-phosphoglycerate kinase | 2.00E-22 |
| **Chaperones** | TC25  (148937) | 4 | Q01899 | HSP70 | HSP70, Hsp70 protein. Hsp70 chaperones help to fold many proteins | 2.00E-06 |
| **Chaperones** | TC58 (CK149023) | 3 | Q01899 | Heat Shock 70KD protein | Hsp70 protein. | 7.00E-108 |
| **Amino acid metabolism** | TC27 (CK149025) | 14 | NP_179032.1 | Putative methylmalonate semi-aldehyde dehydrogenase | NAD-dependent aldehyde dehydrogenases | 7.00E-13 |
| **Translation** | TC31 (CK149041) | 6 | NP_174479.1 | DEAD/DEAH box RNA helicase, putative | SrmB, Superfamily II DNA and RNA helicases | 3.00E-32 |
| **Amino acid metabolism** | TC38 (CK148953) | 4 | F86280 | valyl tRNA synthetase | Valyl-tRNA synthetase | 1.00E-16 |
| **Amino acid metabolism** | TC60 (CK148985) | 4 | NP_179032.1 | putative methylmalonate semi-aldehyde dehydrogenase | Aldehyde dehydrogenase family. | 6.00E-29 |
| **Translation** | TC64 (CK149089) | 3 | NP_174479.1 | DEAD/DEAH box RNA helicase | DEAD/DEAH-box helicases. A diverse family of proteins involved in ATP-dependent RNA unwinding | 6.00E-17 |
| **Amino acid metabolism** | TC70 (CK148894) | 4 | AAN05549.1 | putative valyl tRNA synthetase | ValS, Valyl-tRNA synthetase | 3.00E-49 |
| **Amino acid metabolism** | TC182 (CK148970) | 2 | AAL06923.1 | At4g396601 | BioA, Adenosylmethionine-8-amino-7-oxononanoate aminotransferase | 7.00E-23 |
| **Amino acid metabolism** | TC84 (CK148929) | 4 | AAM83219.1 | At4g03080 | PP2Ac, Protein phosphatase 2A homologues, catalytic domain | 1.00E-18 |
| **Amino acid metabolism** | TC182 (CK148970) | 2 | AAL06923.1 | At4g396601 | BioA, Adenosylmethionine-8-amino-7-oxononanoate aminotransferase | 7.00E-23 |
| **Amino acid metabolism** | TC192 (CK148958) | 7 | NP_201548.1 | formin-like protein | Predicted naringenin-chalcone synthase | 1.00E-09 |
| **Amino acid metabolism** | TC209 (CK148891) | 3 | NP_194437.1 | Tryptophan synthase beta chain 2 | Tryptophan synthase beta chain | 1.00E-82 |
| **Transcription and Translation** | TC91 (CK148967) | 2 | T45677 | ATP dependent RNA helicase | SrmB, Superfamily II DNA and RNA helicases | 2.00E-30 |
| **Transcription and Translation** | TC92 (CK148976) | 5 | CAB75754 | spliceosomal-like protein | CPSF_A, CPSF A subunit region | 5.00E-04 |
| **Transcription and Translation** | TC98 (CK149094) | 5 | AAF34799.1 | 40S ribosomal protein S16 | Ribosomal_S9, Ribosomal protein S9/S16 | 3.00E-26 |
| **Transcription and Translation** | TC185 (CK149116) | 2 | NP_179719.1 | bZIP family transcription factor | BRLZ, basic region leucine zipper | 1.00E-05 |
| **Transcription and Translation** | TC189 (CK149132) | 2 | NP_564034.1 | D3PGD | LOAD_ACT, ACT small ligand binding domain. | 8.00E-04 |
| **Transcription and Translation** | TC205 (CK148949) | 2 | NM117183.2 | RNA directed RNA polymerase | RNA dependent RNA polymerase | 2.00E-13 |
| **Lipid metabolism** | TC187 (CK149076) | 13 | NP_173376.1 | Very-long-chain fatty acid condensing enzyme CUT1 | Predicted naringenin-chalcone synthase | 2.00E-11 |
| **Proteinases and ubiquitin** | TC188 (CK149070) | 2 | T09528 | Probable cysteine proteinase | Papain family cysteine protease | 5.00E-21 |
| **Cell cycle and development** | TC200 (CK148960) | 3 | NP_171799.2 | Putative peroxisome assembly factor-2 | ATPase family associated with various cellular activities (AAA) | 6.00E-04 |
| **Transport** | CK148829 | Singleton | NM_128723 | Putative importin (nuclear transport factor) | SXM1,importin,protein involved in nuclear import) | 4E-9 |

* The longest EST from TCs were submitted to Genbank.
